# South Korea’s COVID-19 Infection Status: From the Perspective of Re-positive Test Results After Viral Clearance Evidenced by Negative Test Results

**DOI:** 10.1017/dmp.2020.168

**Published:** 2020-05-22

**Authors:** Yun-Jung Kang

**Affiliations:** Department of Clinical Laboratory Science, Sang-ji University, Wonju 26339, Republic of Korea

**Keywords:** COVID-19, dead virus, re-positive, social distancing, viral clearance

## Abstract

Coronavirus disease (COVID-19) started to occur in South Korea by means of inflow of the virus from abroad, when a case from Wuhan, China, was first confirmed on January 19, 2020. Although South Korea has drastically reduced the number of new confirmed cases and is stabilizing the situation with its exemplary disease prevention policies, there remains a problem. These are cases that had shown negative results to polymerase chain reaction (PCR) (gene amplification) tests as the COVID-19 virus had become undetectable but turned re-positive after a short period. The Central Clinical Committee determined that these re-positive cases after COVID-19 viral clearance are due to the limits of the test method; it is considered that the genetic material of the “dead virus” remaining in a recovered patient’s body is amplified during the test process. Comprehending the above evidence, re-positive cases of COVID-19 are not infectious; the virus is not even reactivated. However, further research is required as we lack research results on this subject. Until we can be sure, social distancing and other such policies should be maintained.

On December 31, 2019, a severe acute respiratory syndrome, coronavirus 2 (SARS-CoV-2) was first discovered from patients with pneumonia of unknown cause in Wuhan, China. After approximately a month, the virus spread rapidly throughout mainland China.^1^ Later, cases of coronavirus disease 2019, also known as COVID-19, were also confirmed in neighboring Asian countries, including South Korea.^2^ By March 10, 2020, COVID-19’s strong infectivity affected approximately 118,000 patients and killed approximately 4200 people around the world in places such as East Asia, Europe, and North America, causing WHO to declare the situation a pandemic on March 11, 2020.^3^ SARS-CoV-2 is a type of beta-coronavirus originating from animals, with 86.9% genetic homology with bat SARS-like coronavirus.^4^ There are 4 genera in coronaviridae, which include SARS-CoV-2. Among them, coronaviruses of the alpha and beta genera infect humans and other animals. The 229E, OC43, NL63, and HKU types of human coronaviruses are mostly seasonal respiratory viruses causing minor upper airway infection; on the other hand, SARS-coronavirus and Middle East respiratory syndrome (MERS)-coronavirus, which belong to the beta-coronavirus group, result in severe pneumonia.

As noted previously, COVID-19 started to occur in South Korea by means of inflow of the virus from abroad, when a case from Wuhan, China, was first confirmed on January 19, 2020. Since mid-February 2020, mass infection has occurred among religious groups and residents of long-term care facilities in Daegu and Gyeongsangbukdo.^5^ Thus, the South Korean government increased its warning level of infectious disease crisis to “serious” on February 23, 2020.^6^ At this time, South Korea announced its policy of early identification of suspected COVID-19 cases, along with policies of early isolation and treatment. It also promoted national action guides and practiced active prevention, such as social distancing. As a result, the total number of confirmed cases present on April 29 is 10,761, with 8922 (82.9%) among them released from quarantine. With 9 new cases confirmed and 68 new people released from quarantine on April 29, the overall number of patients quarantined has declined. Although South Korea has drastically reduced the number of new confirmed cases and is stabilizing the situation with its exemplary disease prevention policies, there remains a problem. The problem is that cases that had shown negative results to polymerase chain reaction (PCR; gene amplification) tests, indicating the COVID-19 virus was undetectable, upon re-test, turned positive again after a short period of time.

Since the first re-positive case reported on April 8, 2020, the total number of re-positive cases after the release of quarantine at present on April 29 is 292, which is 3.3% of the 8922 patients released (Table 1). The data are from the South Korea Centers for Disease Control and Prevention. The South Korea Centers for Disease Control and Prevention is an organization belonging to the Ministry of Health and Welfare of South Korea. This institution is responsible for the prevention, investigation, quarantine, testing, research, and long-term transplant management of infectious diseases, chronic diseases, rare intractable diseases to improve the national health of South Korea.

**TABLE 1:** Status of Re-positive COVID-19 (00:00, April 29^th^)

Age (y)	No. of Cases	(%)
	Total 292	(100.0)
80 ≤	24	(8.2)
70-79	17	(5.8)
60-69	31	(10.6)
50-59	49	(16.8)
40-49	40	(13.7)
30-39	41	(14.0)
20-29	70	(24.0)
10-19	13	(4.5)
0-9	7	(2.4)

Source: The South Korea Centers for Disease Control and Prevention.

Looking at age groups, patients between 20 and 29 years old occupy the largest proportion of re-positive cases, with 24%. It took an average of 13.5 days (at least 1 day to up to 35 days) to re-positive after being judged negative. The results of clinical and epidemiological information in 292 re-positive cases are asymptomatic or only minor symptoms, such as cough, sputum, fever, and sore throat. On April 17, the Central Disease Control Headquarters organized and distributed Measures for Dealing with Re-positive Cases to manage re-positive patients; it is also planning to complement its management measures according to the research results on tracing contacts to check the cause and infectivity of the disease, along with virus culture tests.^7^ According to current guidelines, the COVID-19 confirmed patient is released from quarantine when all PCR (gene amplification) test results (conducted twice, 24 h apart, after all symptoms, such as fever, have disappeared) are negative. The re-positive patients were isolated, and the virus was again detected in a PCR (gene amplification) test after a very short period of time.

The Central Clinical Committee determined that these re-positive cases after COVID-19 viral clearance are due to the limits of the test method; it is considered that the genetic material of the “dead virus” remaining in the recovered patient’s body is amplified during the test process. In other words, the re-detection of the COVID-19 virus is likely to be the detection of deactivated virus RNA rather than the reactivated or reinfected virus. In South Korea, the PCR (gene amplification) test is used for COVID-19 diagnosis, which amplifies the virus’s gene for detection. The re-positive cases are suspected to be resulting from technical limitations inherent in PCR tests. PCR tests cannot discern if the virus is alive or dead. Moreover, the test results become less reliable when the amount of genetic material of the virus in epithelial cells is small. In addition, the life expectancy of epithelial cells in our respiratory organs is long, with half-life of 3 mo at most. This means that the virus’s RNA in these cells can be detected in PCR tests even 1 or 2 mo after the virus has been eliminated.^8^ The scientific grounds for this argument are as follows: first, as COVID-19 is not a virus causing chronic infection, which goes through a certain length of latent stage after invading the hosts’ cells, its reactivation is not possible from the virological point of view. Second, the chance of re-infection by other coronaviruses is very low, as studies on the human body and other animals show that the immunity in the living organism after the first infection lasts more than 1 y. Third, not 1 case of additional infection by a re-positive case has been reported so far. Furthermore, the cultivation tests turned out all negative when researchers tried to confirm if there is any active virus in the samples of the re-positive cases.

Comprehending the above evidence, re-positive cases of COVID-19 are not infectious; the virus is not even reactivated. However, further research is required as we lack research results on this subject. Until we can be sure, social distancing and other such policies should be maintained.

## Availability of Data and Materials

The datasets used and/or analyzed during the current study are available from the corresponding author on reasonable request.
